# The endovascular performance spectrum of vascular surgery departments in Germany: Results of an online survey among senior department physicians

**DOI:** 10.1007/s00772-016-0157-3

**Published:** 2016-08-03

**Authors:** T. Schmitz-Rixen, G. Torsello, M. Steinbauer, R. T. Grundmann

**Affiliations:** 1 Klinik für Gefäß- und Endovascularchirurgie, Klinikum der Goethe-Universität, Theodor-Stern-Kai-7, 60590 Frankfurt am Main, Deutschland; 2 Klinik für Vaskuläre und Endovaskuläre Chirurgie, Universitätsklinikum und St. Franziskus Hospital Münster, Münster, Deutschland; 3 Klinik für Gefäßchirurgie, Krankenhaus Barmherzige Brüder Regensburg, Regensburg, Deutschland; 4 Deutsches Institut für Gefäßmedizinische Gesundheitsforschung gGmbH, Berlin, Deutschland

**Keywords:** Vascular surgery, Endovascular surgery, Specialist, Training, Survey, Gefäßchirurgie, Endovaskuläre Chirurgie, Spezialist, Training, Erhebung

## Abstract

**Aim:**

To survey the scope of vascular surgery services in Germany.

**Method:**

A total of 308 senior German vascular surgeons received a 19-point questionnaire pertaining to department structure and scope of services. Of these surgeons 223 replied between 16 August 2015 and 23 October 2015 (response rate 72 %), with 62.2 % reporting an additional qualification as an endovascular surgeon according to the guidelines of the German Society for Vascular Surgery and Vascular Medicine (Deutsche Gesellschaft für Gefäßchirurgie und Gefäßmedizin, DGG) and 43.5 % as a DGG® endovascular specialist.

**Results:**

The number of respondents fully authorized to train in vascular surgery was 71.3 %, while 28.3 % were authorized for limited training. Authorization as a DGG® endovascular surgeon was reported by 24.2 % and authorization as a DGG® endovascular specialist by 17 % of respondents. All respondents performed endovascular interventions on pelvic vessels and 99.1 % also reported carrying out femoral and popliteal endovascular interventions. Endovascular procedures in crural vessels were carried out by 90.1 % and 93.7 % of vascular surgeons performed endovascular procedures in the region of the abdominal aorta (segment V), arteriovenous (AV) fistulas and shunts (85.2 %), upper extremity vessels (80.3 %), the thoracic aorta (segment III, 68.2 %), renal arteries (62.8 %) and visceral aorta (segment IV, 60.5 %). In all 43.5 % of respondents reported experience with endovascular procedures on the carotid bifurcation. Percutaneous arterial procedures formed the focus of endovascular activity, totalling on average 259 interventions per year and department, followed by diagnostic angiography (without intervention) at 166 procedures per year and hybrid arterial interventions at 141 interventions per year.

**Conclusion:**

This survey revealed a high level of endovascular expertise among vascular surgeons in Germany. This applies not only to the scope of endovascular activities in diagnosis and treatment but also to the number of estimated annual procedures.

## Zusammenfassung

**Zielsetzung:**

In einer Umfrage sollte das Leistungsspektrum der deutschen Gefäßchirurgie ermittelt werden.

**Methodik:**

In der Zeit vom 16. August 2015 bis 23. Oktober 2015 wurden insgesamt 308 leitende Gefäßchirurgen zu ihrer Abteilungsstruktur und ihrem Leistungsspektrum anhand von 19 Punkten befragt. Geantwortet haben 223 (Beantwortungsquote 72 %). Eine Zusatzqualifikation *Endovasculärer Chirurg DGG*® nannten 62,2 %, eine Qualifikation *Endovasculärer Spezialist DGG*® 43,5 %.

**Ergebnisse:**

Insgesamt 71,3 % gaben eine volle Ermächtigung zur Weiterbildung in der Gefäßchirurgie an, 28,3 % eine Ermächtigung zur Teil-Weiterbildung Gefäßchirurgie. Eine Ermächtigung zum endovaskulären Chirurgen DGG® meldeten 24,2 %, eine solche zum endovaskulären Spezialisten DGG® 17 % der Antwortenden. Alle Antwortenden berichteten, an Beckengefäßen und 99,1 % an Oberschenkelstrombahn und A. poplitea endovaskulär therapeutisch tätig zu sein, an den Unterschenkelgefäßen waren es 90,1 %. Sehr hoch war auch der Prozentsatz an Gefäßchirurgen, die im Bereich der abdominellen Aorta (Abschnitt V; 93,7 %), an AV-Fisteln und Shunts (85,2 %), Armgefäßen (80,3 %), thorakaler Aorta (Abschnitt III; 68,2 %), Nierenarterien (62,8 %) und viszeraler Aorta (Abschnitt IV; 60,5 %) endovaskuläre Eingriffe vornahmen. Erfahrung mit endovaskulären Eingriffen an der Karotisgabel nannten 43,5 % der Antwortenden. Den Schwerpunkt der endovaskulären Tätigkeit machten perkutane Interventionen an den Arterien aus, von denen durchschnittlich 259 Eingriffe pro Jahr und Abteilung vorgenommen wurden. Es folgten die diagnostischen Angiographien (ohne Intervention) mit 166 jährlichen Prozeduren und Hybrideingriffe an den Arterien mit 141 jährlichen Interventionen.

**Folgerung:**

Unsere Umfrage zeigt die hohe Kompetenz der Gefäßchirurgen bei endovaskulären Eingriffen in Deutschland. Dies gilt sowohl für den Umfang der endovaskulären Tätigkeit in Diagnostik und Therapie als auch die Zahl der geschätzten jährlich durchgeführten Eingriffe.

## Background and objective

Endovascular interventions have become part of the standard repertoire of vascular surgeons. For example, Sachs et al. [1] identified 563,143 patients in the nationwide inpatient sample (NIS), representing a sample of approximately 20 % of all hospitalizations in the USA who underwent interventions for intermittent claudication (IC) or critical limb ischemia (CLI) in the period between 1999 and 2007. Of these patients 218,655 (38.8 %) were treated by an endovascular procedure, i.e. percutaneous transluminal angioplasty (PTA) and stent, 280,021 underwent peripheral bypass graft (49.7 %), 36,307 (6.4 %) aortofemoral bypass and 5.1 % a hybrid procedure. The CRITISCH study [2], which was supported by the German Society for Vascular Surgery and Vascular Medicine (Deutsche Gesellschaft für Gefäßchirurgie und Gefäßmedizin, DGG), recently showed that endovascular treatment was the preferred approach in CLI, representing the treatment of first choice in 53.4 % of cases, followed by bypass surgery in 23.7 %. Finally, the endovascular approach plays a particularly important role in the treatment of abdominal aortic aneurysms (AAA). On the basis of the Medicare database (patients aged 67 years or older) for the period 2001–2008, Schermerhorn et al. [3] reported 128,598 patients who received elective treatment for AAA, 79,463 of whom underwent endovascular repair (61.8 %) and 49,135 open repair (OR). The percentage of endovascular procedures for the treatment of intact AAA (iAAA) has meanwhile continued to rise, totalling 72 % in the German Institute for Vascular Medicine Healthcare Research [4] (Deutsches Institut für Gefäßmedizinische Gesundheitsforschung, DIGG) register for 2014. Diagnosis-related groups hospital statistics released by the German Federal Statistical Office (Statistisches Bundesamt) permit an overview of inpatient endovascular treatment in Germany [5]. Table 1 provides some information on the procedures carried out in 2013. The statistics for 2013 also show 40,668 arteriograms of neck vessels, 77,140 of abdominal vessels, 138,860 of pelvic vessels, 159,990 of lower extremity vessels and 43,668 super-selective arteriograms, excluding coronary angiograms.

**Table 1:** Endovascular procedures in German hospitals in 2013 (Source: German Federal Statistical Office 2014)

Procedure	German operation and procedure code	Number (*n*)
Percutaneous transluminal vascular intervention, extracranial internal carotid artery	8-836.0k	2427
Percutaneous transluminal vascular intervention, extracranial internal carotid artery and common carotid artery	8-836.0m	2611
*Abdominal aorta*		
Bifurcation graft, aorto-bi-iliac without fenestration or side branch	5-38a.14	7082
Tubular graft, iliac without sidearm	5-38a.40	1812
Tubular graft, aortic without fenestration or sidearm	5-38a.1e	809
*Open surgical angioplasty (balloon)*		
Other abdominal and pelvic vessels	5-38f.9	3783
Open surgical angioplasty: femoral vessels	5-38f.b	5107
Open surgical angioplasty: lower leg vessels	5-38f.c	2318
*Percutaneous transluminal vascular interventions*		
PTA: other abdominal and pelvic vessels	8-836.09	28,489
PTA: visceral vessels	8-836.0a	3747
PTA: femoral vessels	8-836.0b	67,048
PTA: lower leg vessels	8-836.0c	38,792

*PTA* percutaneous transluminal angioplasty

We conducted a survey to make a better assessment of the scope of services offered by vascular surgeons in Germany specifically in relation to endovascular treatment and the results of this survey are presented. At the same time, we have used the subject as an opportunity to provide an overview of the numbers reported for endovascular training in the literature.

## Methods

**Fig. 1:**
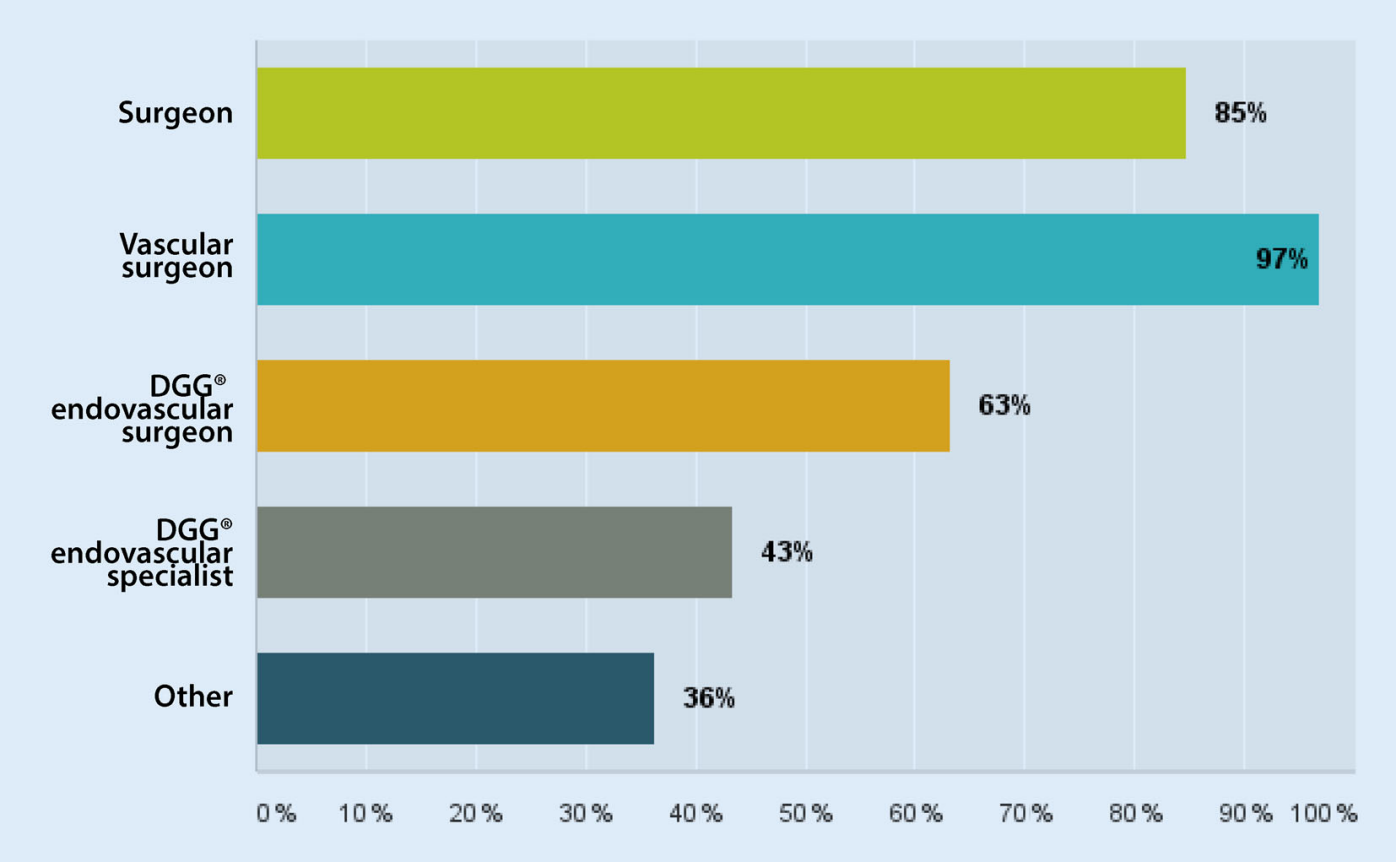
Further training status among senior physicians

Between 16 August and 23 October 2015 a total of 308 senior vascular surgeons were surveyed on departmental structure and scope of services using a 19-point questionnaire. To this end, more than 95 % of all senior departmental heads of vascular surgical units in Germany gained access to an online questionnaire. By checking and blocking IP addresses once questionnaires had been completed, it was largely possible to make access selective and preclude the possibility of responding twice. Responses were received from 223 vascular surgeons in total, representing a response rate of 72 %. The following information on questions and answers relates to this collective. In all 216 out of 223 respondents (96.8 %) held the qualification of vascular surgeons and 189 (84.8 %) the qualification of surgeon (Fig. 1). Of these 62.2 % reported an additional qualification as a DGG® endovascular surgeon and 43.5 % as a DGG® endovascular specialist. A total of 78.9 % had gained specialist knowledge of interventional radiology, 72.6 % of radiology and 10.7 % of computed tomography (CT) radiology. Of the respondents 30 (13.5 %) worked in university hospitals, 39 (17.5 %) in maximum care institutions, 83 (37.2 %) in specialized care institutions, 64 (28.7 %) in basic and standard care institutions, 5 (2.2 %) in specialist hospitals, 1 in an external physician department, and 1 in private practice.

In terms of departmental structure, 171 (76.7 %) specified a vascular surgery department, 47 (21.1 %) a unit within a general surgical department, and 5 (2.2 %) a unit within a cardiac surgery department. The number of staff per department was reported to include on average one chief physician, three senior physicians, one specialist vascular surgeon with no official position and three assistant physicians. Of the centers surveyed 57 % had no certification, 24 % were certified by the DGG alone, 12 % had triple certification and 7 % double certification. Table 2 lists the technical facilities available in the surveyed departments.

**Table 2:** Technical facilities in vascular surgery departments

Response options	Number of responses (*n*)	%
C-arm	135/223	60.5
C-arm with flat detector	99/223	44.4
Hybrid procedure	75/223	33.6
Mini-hybrid procedure	10/223	4.5
Angio-suite	91/223	40.8

## Results

### Scope of endovascular activities: diagnosis

In terms of diagnosis a distinction was made in the questionnaire between angiography, shuntography and diagnostic phlebography. Of the 223 respondents 187 (83.9 %) reported performing diagnostic angiography, 176 (79.9 %) shuntography and 85 (38.1 %) diagnostic phlebography.

### Scope of endovascular treatment activities

**Fig. 2:**
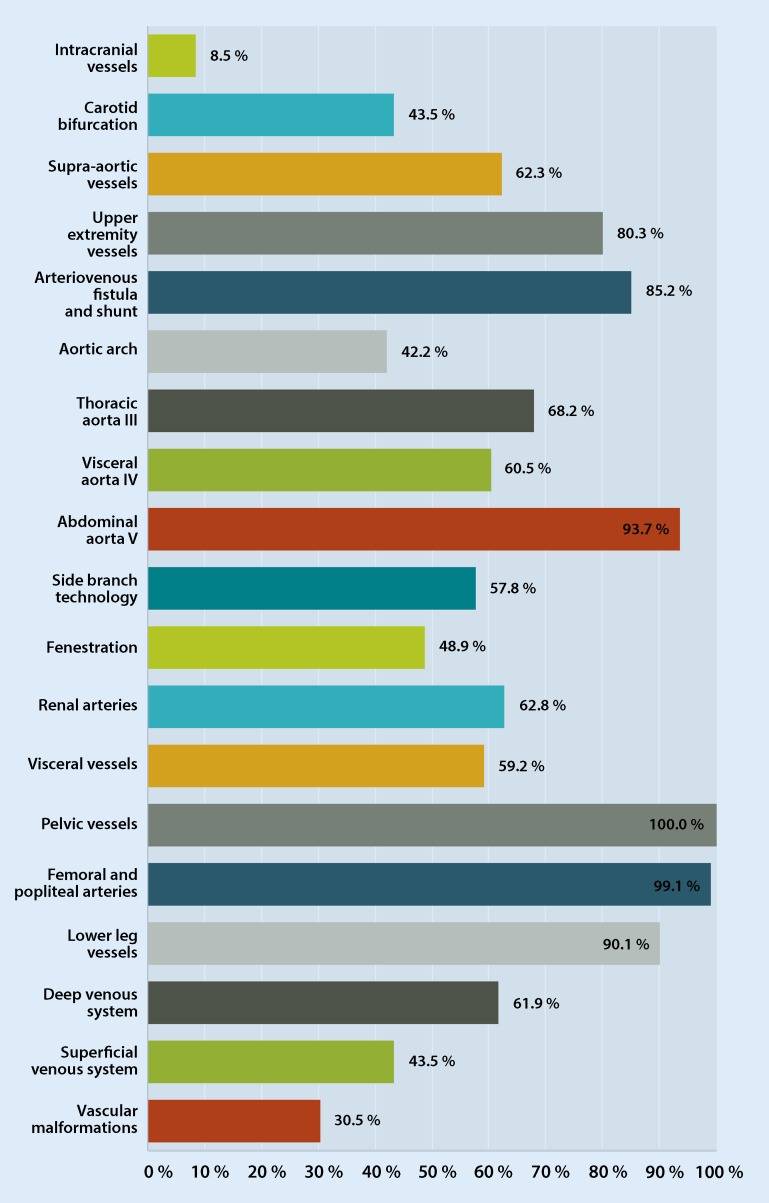
Scope of endovascular treatment activities

Fig. 2 shows the scope of endovascular treatment activities in detail. All respondents reported performing endovascular interventions on pelvic vessels, 99.1 % on femoral and popliteal arteries and 90.1 % on crural vessels. A high percentage of vascular surgeons performed endovascular interventions in the abdominal aortic region (segment V, 93.7 %), arteriovenous (AV) fistulas and shunts (85.2 %), upper extremity vessels (80.3 %), the thoracic aorta (segment III, 68.2 %), renal arteries (62.8 %) and the visceral aorta (segment IV, 60.5 %). Of the respondents 43.5 % reported experience with endovascular procedures on the carotid bifurcation. Only experiences with vascular malformations (30.5 % of respondents) and endovascular intracranial procedures (8.5 %) were reported comparatively rarely. When asked whether endovascular procedures involved hybrid or percutaneous approaches 99.1 % reported performing hybrid and 71.3 % percutaneous interventions.

### Reported number of annual endovascular interventions

The reported numbers of procedures performed annually per department (based either on figures for 2014 or projected figures for 2015) are given in Table 3. Percutaneous arterial interventions represented the main focus of endovascular activities, with 259 interventions performed on average per year and per responding department. These were followed by diagnostic angiograms (without intervention) at 166 procedures per year and hybrid arterial interventions at 141 interventions per year.

**Table 3:** Annual number of procedures performed

Response options	Average number per responding department	Total number (*n*)	Number of respondents
Diagnostic angiograms	166	32,575	196
Percutaneous arterial interventions	259	51,078	197
Percutaneous interventions on deep veins	9	1557	173
Percutaneous interventions on the superficial venous system	45	7254	160
Hybrid arterial procedures	141	30,988	220
Hybrid venous procedures	8	1295	160
Interventions on dialysis shunts	35	7080	204
Interventions for vascular malformations	5	717	149
Others	24	844	35

### Departments performing activities

#### Diagnostic angiography

The questionnaire asked which department in the hospital most commonly performed diagnostic angiography. Of the respondents 14.4 % reported that 51–75 % of diagnostic angiograms were performed in the radiology department, a further 34 % reported corresponding percentages of 76–99 % and 29.4 % stated that all diagnostic angiograms were performed in the radiology department. A total of 14.6 % respondents stated that all diagnostic angiograms were performed in the vascular surgical unit. In contrast, 17.4 % stated that no diagnostic angiograms were performed in the vascular surgical unit, while a further 45.5 % put the percentage of vascular surgical unit-based diagnostic angiograms as a percentage of all diagnostic angiograms at 1–25 %.

#### Percutaneous arterial procedures

**Fig. 3:**
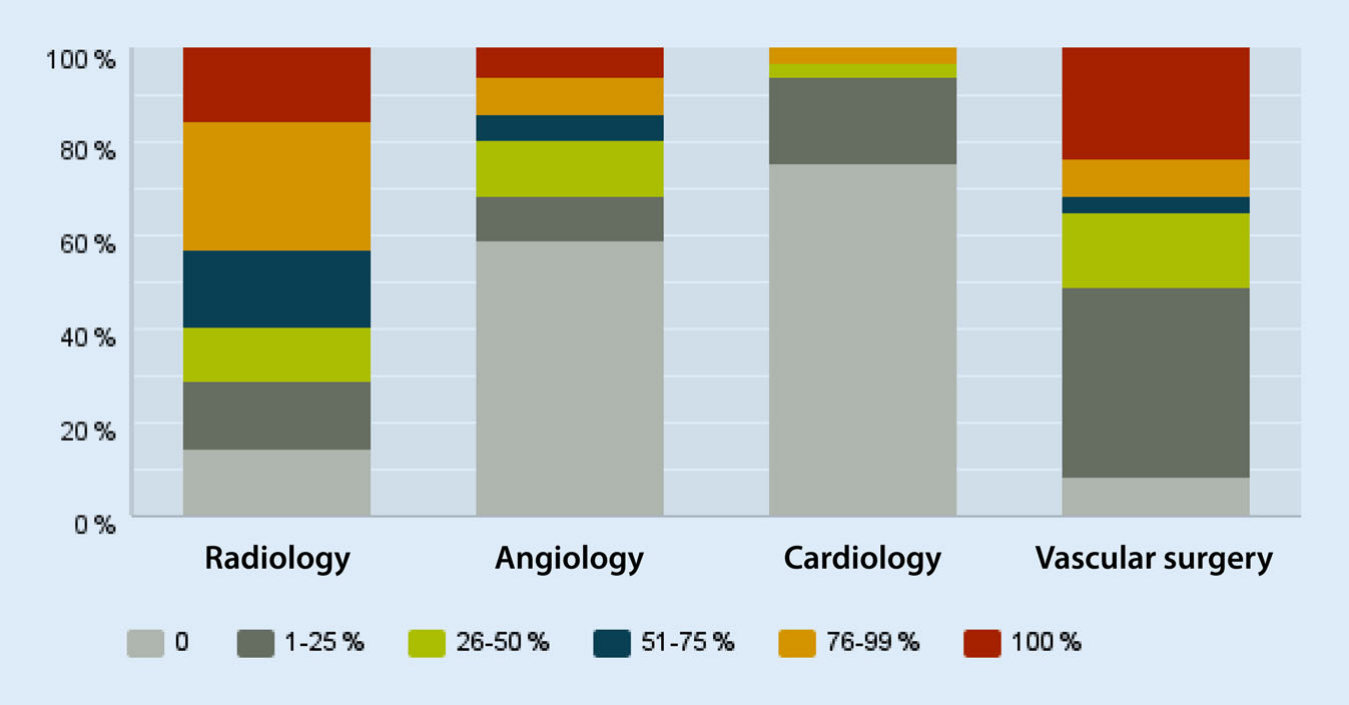
Percentage share of the various departments of the total volume of percutaneous arterial interventions

The percentage of percutaneous arterial interventions performed in the radiology department was put at 51–75 % by 16.7 % of respondents, at 76–99 % by 27.4 %, and at 100 % by 15.5 %. A total of 14.5 % reported that no percutaneous arterial interventions were performed in the radiology department (Fig. 3).

With regard to vascular surgical departments, 8.3 % reported percentages of 76–99 % and 23.3 % a percentage of 100 %. In contrast, 8.2 % stated that no percutaneous arterial interventions were performed in vascular surgical departments, while 40.9 % gave corresponding percentages of 1–25 %. Percutaneous arterial interventions were relatively rare in angiology and cardiology departments. For example, 58.7 % stated that no interventions were performed in the angiology department at their institution. In contrast, only 7.8 % and 6.1 % reported that 76–99 % or indeed all of percutaneous arterial interventions were performed in angiology departments. Altogether, 75.5 % reported that no percutaneous arterial interventions were performed in cardiology departments. Only 3 % put the proportion of percutaneous arterial interventions performed in cardiology departments at 76–99 %.

#### Percutaneous venous procedures

In total, 43.9 % reported that all venous percutaneous procedures were performed in vascular surgical departments, while 7.3 % put this at 76–99 %. That no venous procedures were performed in the vascular surgical department was reported by 23.1 %, while a further 10.9 % put this at 1–25 %. Comparative figures for radiology departments were as follows: 33.1 % reported that all percutaneous venous procedures were performed in radiology departments, while 9 % put this at 76–99 %. In all, 32.4 % reported that no venous procedures were performed in radiology departments and 11 % that 1–25 % were performed. With regard to angiology departments, 78.6 % stated that no percutaneous venous procedures were performed here and the corresponding figure for cardiology departments was 95.5 %.

#### Procedures for malformations

More than 75 % of malformations were treated exclusively in vascular surgical departments, the remainder primarily in radiology departments.

#### Endovascular component of the hybrid procedure

**Fig. 4:**
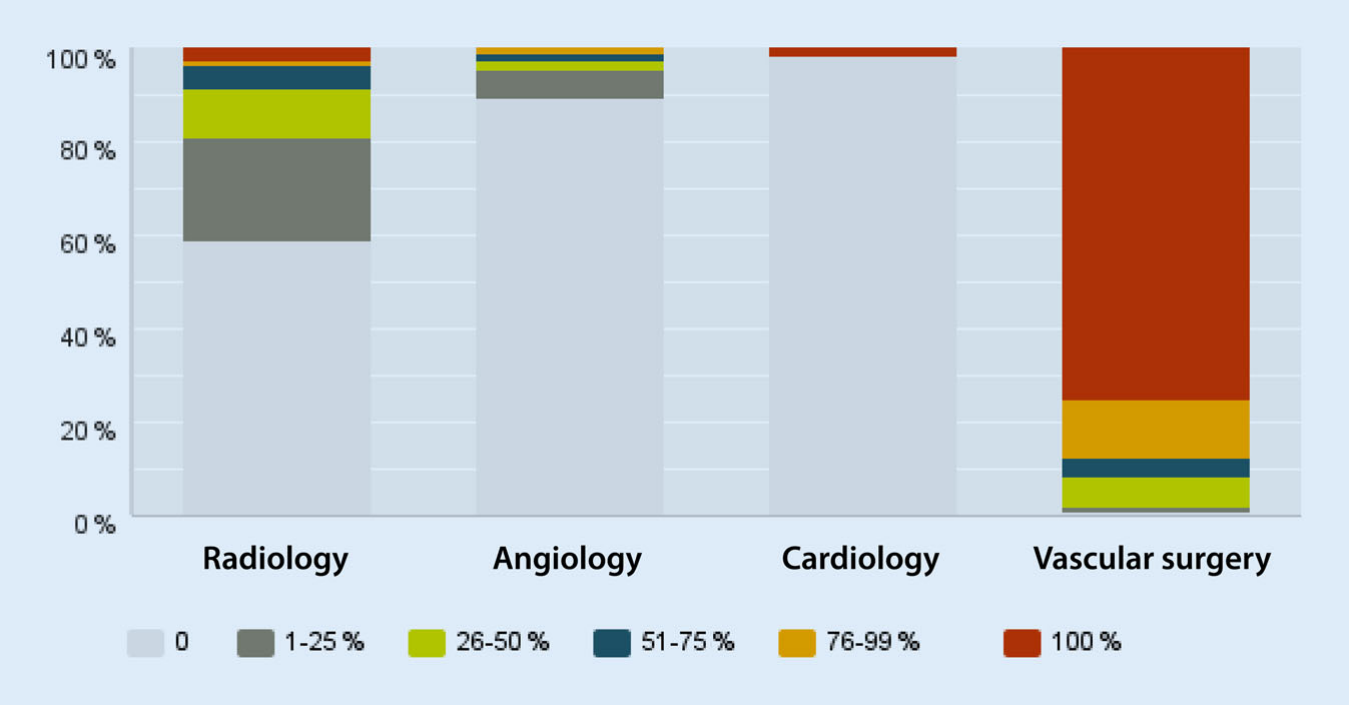
Percentage share of the various departments of the total volume of hybrid procedures

Respondents were asked who performed the endovascular component of hybrid procedures. In 75 % of departments, this person was solely the vascular surgeon and in other 25 % by the radiologist. Altogether, 58.9 % of radiologists, 89.7 % of angiologists and 98.8 % of cardiologists in the departments surveyed had no involvement whatsoever in hybrid procedures (Fig. 4).

### Authorization to provide further training

At total of 71.3 % of respondents were fully authorized to provide vascular surgical training and 28.3 % to provide limited training. Authorization to provide training to DGG® endovascular surgeon level was reported by 24.2 % and to DGG® endovascular specialist level by 17 % of respondents. Further training in endovascular techniques was provided by vascular surgeons in 96.8 % of the departments surveyed, radiologists in 36.3 %, angiologists in 9 %, and cardiologists in 2.2 %.

#### Guest students

Respondents were also asked whether their departments had capacity to supervise the further training of guest students and if yes, how many guest students and for what period of time? Altogether, 95 departments were able to take guest students for 1 month, 78 for 3 months, 43 for 6 months and 64 for over 6 months. Thus, at least 104 guest students could be supervised for 1 month, 88 for 3 months, 51 for 6 months, and 88 for even longer than 6 months.

## Discussion

The results of the survey revealed a high level of endovascular expertise among vascular surgeons in Germany. This applied not only to the scope of endovascular activities in diagnosis and treatment but also to the number of estimated annual procedures. Nowadays, experience in endovascular techniques is required of all vascular surgeons working in western countries [6]. In a survey of members of the Society for Vascular Surgery (SVS) conducted by Matthews et al. [7], 37.8 % of respondents reported that endovascular procedures accounted for 51–75 % of the case volume. Of these respondents, 20 % estimated that they performed open procedures as frequently as endovascular procedures and only 20 % reported that endovascular interventions accounted for less than 25 % of the activities. As many as 12 % of respondents stated that endovascular procedures accounted for even more than 75 % of the case volume. An age dependence was seen in this survey: 89.9 % of younger vascular surgeons (aged below 50 years) reported that endovascular procedures made up half of the workload, while this proportion was reported by only 62 % of vascular surgeons aged over 50 years. Harkin et al. [8] surveyed 450 vascular surgeons in the UK on their activities and 352 surgeons responded (78 % response rate). Over 90 % of vascular surgeons performed the index operations open aneurysm repair, carotid endarterectomy, infrainguinal bypass and amputation, while 84 % performed standard endovascular aortic repair (EVAR). More complex endovascular procedures, such as thoracic EVAR (TEVAR, 39 %), fenestrated EVAR (35 %) and branched EVAR (24 %) were performed less frequently. Peripheral angioplasty belonged to the repertoire of 31 % of vascular surgeons. Only 20 % of surgeons performed complex open thoracoabdominal aortic surgery and 43 % renal access surgery. The lower density of vascular surgeons in the UK (7 surgeons per 1 million population at the time of the survey) needs to be taken into account in the description of this field of activity.

Endovascular interventions in the lower extremities are performed by a number of different specialists. Wallace et al. [9] analyzed routine data from the Florida Agency for Health Care Administration including 15,398 hospitalized patients between 2005 and 2009 not undergoing concomitant surgical procedures. The percentage of procedures performed by vascular surgeons from all endovascular interventions rose steadily during this period from 28 % to 48 % in 2009, while the percentage of interventional cardiologists dropped from 47 % to 33 % and interventional radiologists from 25 % to 20 %; however, as the majority of these interventions are performed in an outpatient setting in the USA, the quota of individual disciplines in relation to the total case volume should be estimated with caution. A study conducted by Harris et al. [10] based on data from the Centers for Medicare & Medicaid Services (CMS) in the USA is potentially better suited to this end. The CMS database contained all procedure-related invoices for this patient population (patients over 65 years). According to this study, growth was seen in all disciplines (i.e. radiology, cardiology and vascular surgery) for the code 37205 (transcatheter placement of an intravascular stent in a vessel, excluding coronary, carotid and vertebral arteries) between 2000 and 2007, the highest growth being seen in vascular surgery; however, the percentage performed by vascular surgeons was still smaller than those performed by radiologists and cardiologists. Calculated on the basis of 100,000 Medicare insurees, vascular surgeons performed 54 of these interventions in 2007, cardiologists 95 and radiologists 67. The highest growth in vascular surgery was also registered for the code 75962 (transluminal balloon angioplasty, peripheral arteries), whereby the percentage performed by vascular surgeons went on to exceed that of radiologists: looking at a population of 100,000 Medicare insurees, 64 of these interventions were carried out by vascular surgeons, 78 by cardiologists and 58 by radiologists in 2007.

The study by Wallace et al. [9] highlighted that vascular surgeons dealt with the more serious cases, treating 50 % of all patients with CLI, while interventional cardiologists, in contrast, treated 57 % of all patients with IC. This confirms the results of an earlier study by Vogel et al. [11] who analyzed a total of 1887 percutaneous transluminal angioplasty (PTA) on lower extremities, 1021 performed by vascular surgeons and 866 by cardiologists. Here again, the vascular surgical patients were significantly more severely diseased. The percentage of patients with IC in the vascular surgical patient population was 60.7 % but 80.7 % in the cardiology population. In contrast, patients with at resting pain accounted for 16.0 % of vascular surgical patients and gangrene patients for 23.3 %. The corresponding figures for cardiologists were 6.2 % and 13.1 %. Vogel et al. [11] also calculated the cost of resources under North American billing conditions. According to their calculation, treatment in vascular surgical departments was significantly more cost-effective than in cardiology departments but with comparable outcomes. Reasons for this included the fact that vascular surgeons utilized fewer resources and hence worked more cost-effectively compared with cardiologists (in terms of the number of catheters used and technical equipment/medical supplies/number of consultations). Particularly with regard to EVAR for AAA, the leading expertise of the vascular surgeon is undisputed. This was demonstrated by an analysis of the NIS in the USA for the period 2001–2009 that included 28,094 EVAR procedures, 97 % of which were for non-ruptured AAA [12]. In this collective, 92.2 % of all EVAR procedures were performed by surgeons (not further differentiated) and 7.8 % by interventionalists (also not further differentiated). Patients treated by surgeons exhibited lower hospital mortality (2.0 % vs. 4.3 %) and, as a gauge for complications, shorter average hospital stays (3.69 vs. 5.88 days); however, this benefit for surgery did not reach clinical significance when only therapists with large case volumes were compared.

## Further endovascular training

A survey conducted by Dalsing et al. [13] of vascular surgery residents in training demonstrated where the focus of interest lies among trainee vascular surgeons in the USA today: the teaching faculty and endovascular facilities were the most important factors in their ranking of a training program, with 68.8 % and 60 %, respectively, rating these as very important. These were followed by case volumes in open aortic surgery (43.8 %), open carotid surgery (40.0 %) and thoracic aortic interventions (37.6 %) in the ranking of importance for the choice of training program. Residents were also asked about their levels of competence (self-grading) and graded own endovascular competence as high with 78.1 % who considered themselves very competent in general endovascular techniques and 68.8 % in EVAR and TEVAR; however, only 38.7 % considered themselves highly competent in carotid stenting and familiarity with lytic therapy was also lower at 56.3 %. The number who graded themselves as very competent in carotid endarterectomy (CEA) was 78.1 %, in lower extremity revascularization 71.9 % and in open abdominal aortic surgery 46.9 %. How the scope of vascular surgical practice has changed in recent decades was strikingly demonstrated by a comparison of the 10 most common procedures reported by applicants for the vascular surgery recertification examination in the USA in 1995 vs. 2009 ([14]; Table 4).

**Table 4:** The 10 procedures most frequently reported by US vascular surgeons applying for recertification examination in 1995 vs. 2009 (adapted from Eidt et al. [14])

Rank	1995	2009
1	Leg bypass (all)	Arteriogram
2	CEA	Varicose vein
3	AV graft	PTA
4	AAA (open)	Stent
5	Thrombectomy	Leg bypass (all)
6	Varicose vein	AV fistula
7	Digit amputation	CEA
8	Below-knee amputation	Venogram
9	AV fistula	IVC filter
10	IVC filter	EVAR

*CEA* – carotid endarterectomy, *AV* – arteriovenous, *PTA* – percutaneous transluminal angioplasty, *AAA* – abdominal aortic aneurysm, *IVC* – inferior vena cava, *EVAR* – endovascular aortic repair

The average number of cases submitted by applicants in 1995 was 183 and 647 in 2009

In 2010 Fitridge et al. [15] proposed an international core curriculum for endovascular surgical training, which set 100 diagnostic angiograms, 50 angioplasties/stents (20 aortoiliac, 15 femoral above-knee popliteal and 15 infragenicular), as well as 20 EVAR as the minimal requirements for specialist recognition. Can a trainee vascular surgeon achieve this case log on a 1-2 year training program and where does the focus lie? Schanzer et al. [16] sought answers to these questions and according to their analysis, open procedures accounted for approximately 48 %, diagnostic endovascular procedures 20 % and therapeutic endovascular procedures 32 % of the 519 major vascular surgical procedures performed on average by graduating trainees in 2007. Approximately 50 EVAR were set against 41 open aortic procedures, 42 CEA, and 36 open peripheral bypasses, to mention the most common. Schanzer et al. [16] found a significant increase in the mean number of procedures performed by trainees between 2001 and 2007, from 298 to 519, attributable almost entirely to the substantially increased case volume in 2007 compared with 2001. Parallel to this increase in endovascular procedures, only 65 % of trainee vascular surgeons questioned in another US survey believed that they would leave their training program with competence levels that prepared them well for open vascular surgery in their future practice, whereas 84 % felt confident that they could provide the full range of endovascular surgical procedures [17].

With their survey Reed et al. [18] set out to ascertain the extent to which vascular surgical departments in the USA have the capacity to adequately train vascular surgery resident trainees in interventional techniques. In all 191 out of 240 trainees who had been trained in 91 hospitals with special training programs responded. In 2008 it was possible to learn interventional techniques largely (over 80 %) in vascular surgical institutions, most commonly with EVAR (99 %), suprainguinal (94.8 %) and infrainguinal (92.7 %) PTA/stenting and least commonly with carotid artery stenting (CAS, 82.2 %). Trainees additionally received training in interventional techniques in cardiology (more rarely) and interventional radiology (more frequently). When asked about the percentage accounted for by the individual departments in terms of their training in interventional techniques, 38.5 % of respondents estimated that 75–100 % of their training had been obtained in the vascular surgical department, 30.8 % put this figure at 51–75 % and a further 30.8 % at 26–50 %. A UK survey of 217 trainee vascular surgeons, of which 153 (71 %) responded, painted a very different picture [19]. At the time of the survey 80 of these 153 (52 %) were working in posts that offered no endovascular training and 88 % (123/153) had performed less than 10 peripheral angiograms and/or angioplasties with or without supervision in the previous 12 months. A total of 63 % (96/153) reported that they had never performed a procedure of this kind. The majority of trainees (104/153) had performed less thatn10 EVAR (partially or completely, with or without supervision) in the previous 12 months. Furthermore, no experience with endovenous laser therapy, radiofrequency ablation or foam sclerotherapy for varicose vein treatment was reported by 33 %, 49 %, and 46 %, respectively. This survey demonstrates, in terms of individual vascular surgical department capacity to offer further training, the range of endovascular services available and the opportunities offered to endovascular guest students, among other factors that at least endovascular training opportunities in Germany for trainee vascular surgeons in vascular surgical departments are significantly broader. Finally, a further and particular aspect of endovascular activity requires mention, i. e. radiation exposure. Kirkwood et al. [20] showed that providing surgeons with training in techniques aimed at reducing radiation exposure during endovascular procedures is an important factor in reducing radiation doses in the interventional context. In our survey 72.6 % of respondents reported having specialist knowledge of radiology, while 78.9 % reported specialist knowledge of interventional radiology. To what extent these rates can be improved on remains open.

## Conclusion

- At a response rate of 72 %, the present survey can be considered representative.
- The data show high levels of competence in endovascular procedures among vascular surgeons in Germany.
- This applies not only to the scope of diagnostic and therapeutic endovascular activities but also to the number of estimated annual procedures performed.
